# Processing Preference Toward Object-Extracted Relative Clauses in Mandarin Chinese by L1 and L2 Speakers: An Eye-Tracking Study

**DOI:** 10.3389/fpsyg.2016.00004

**Published:** 2016-01-21

**Authors:** Yao-Ting Sung, Jung-Yueh Tu, Jih-Ho Cha, Ming-Da Wu

**Affiliations:** ^1^Department of Educational Psychology and Counseling, National Taiwan Normal UniversityTaipei, Taiwan; ^2^Center of Learning Technology for Chinese, National Taiwan Normal UniversityTaipei, Taiwan; ^3^International Chinese Education Center, School of Humanities, Shanghai Jiao Tong UniversityShanghai, China

**Keywords:** relative clauses, Mandarin Chinese, L2 sentence processing, eye-movements, Japanese CSL learners

## Abstract

The current study employed an eye-movement technique with an attempt to explore the reading patterns for the two types of Chinese relative clauses, subject-extracted relative clauses (SRCs) and object-extracted relative clauses (ORCs), by native speakers (L1), and Japanese learners (L2) of Chinese. The data were analyzed in terms of gaze duration, regression path duration, and regression rate on the two critical regions, head noun, and embedded verb. The results indicated that both the L1 and L2 participants spent less time on the head nouns in ORCs than in SRCs. Also, the L2 participants spent less time on the embedded verbs in ORCs than in SRCs and their regression rate for embedded verbs was generally lower in ORCs than in SRC. The findings showed that the participants experienced less processing difficulty in ORCs than SRCs. These results suggest an ORC preference in L1 and L2 speakers of Chinese, which provides evidence in support of linear distance hypothesis and implies that the syntactic nature of Chinese is at play in the RC processing.

## Introduction

Relative clauses (RCs) have received considerable attention in psycholinguistic and linguistic research over the past few decades. An RC is a subordinate clause that modifies a noun and is embedded within a noun phrase. There are two major types of RCs: subject-extracted relative clauses (SRCs) and object-extracted relative clauses (ORCs). Examples of an SRC and an ORC in English are given in (1a) and (1b), respectively.

(1a)     English SRC
             The     principal*_*i*_*     [who     *e_*i*_*     introduced     the teacher]_RC_     talked     in a very polite manner
             FILLER                 REL     GAP
             “The principal who introduced the teacher talked in a very polite manner.”
(1b)     English ORC
              The     principal*_*i*_*     [who     the teacher     introduced     *e_*i*_*]_RC_     talked     in a very polite manner
              FILLER                 REL                                                    GAP
              “The teacher whom the principal introduced talked in a very polite manner.”

In (1), “the principal” is extracted from the clause and leaves an empty position, which is called a *gap*. The relative pronoun “who” introduces the RC. The extracted noun phrase “the principal” is coindexed with the gap and is called the *filler*, because it should fill the gap. The two types of RCs only contrast each other with respect to the location of the gap. Hence, comprehending and integrating RCs requires dependency between the filler and gap to be developed in harmony.

SRCs are considered easier to process than ORCs, with the evidence coming from observations of RC processing by native speakers (L1) of head-initial languages (e.g., for English, see Gordon et al., 2006; for Dutch, see Frazier, 1987; for French, see Holmes and O'Regan, 1981; for German, see Schriefers et al., 1995). The SRC preference is also evident in head-final languages (e.g., for Japanese, see Ueno and Garnsey, 2008; for Korean, see Kwon et al., 2010). In addition, it is reported in studies on second language (L2) comprehension (Gass, 1979; Doughty, 1991; Hamilton, 1994). The findings of these studies have led to the conclusion that SRCs are easier to process cross-linguistically than ORCs both in L1 and L2 sentence processing. However, conflicting results have been reported on the processing-difficulty contrast between SRCs and ORCs in Mandarin Chinese (Chinese, hereafter; Chen et al., 2012). Reports of the ORC processing preference in Chinese^1^ (e.g., Hsiao and Gibson, 2003; Hsu and Chen, 2007; Lin and Garnsey, 2011; Gibson and Wu, 2013; Sung et al., 2015) have posited a challenge to the presence of a universal SRC processing preference.

Previous studies (e.g., Hsiao and Gibson, 2003; Gibson and Wu, 2013) were conducted using self-paced reading tasks. This method usually requires readers to press the button for the occurrence of each word, which causes repeated interruptions in reading. The self-paced reading task cannot completely record or reflect the normal reading process, wherein readers can move back and forth within a sentence, such as using regression or saccades. It also limits the scope of research and lacks certain online processing information. In particular, reading RC sentences requires dependency between the gap and filler so readers may read back and forth for integration and comprehension, which can be obtained with an eye-tracking device.

The goal of this study is to re-examine the L1 processing of Chinese RCs and further expand it to L2 RC processing. Since Chinese is a head-initial language with a head-final RC pattern while Japanese is a head-final language and the two languages share some syntactic similarities, such as RC location (both prenominal) and gap position (both prenominal), it would be interesting to see how the Japanese speakers, whose language has a typologically different RC structure, process Chinese RCs. Specifically, the study intends to see whether the Chinese syntactic nature or the universal SRC preference plays a more crucial role in the L2 RC processing by Japanese speakers. The native speakers of Chinese were also manipulated in the experiment. This study would like to serve as a pioneer eye-tracking research on L2 RC processing, which invites further studies on L2 learners with different language profiles.

### Aspects of chinese and japanese RC

#### Chinese RC structure and its processing

The structure of a Chinese RC is different from that of an English RC. In Chinese, an RC precedes the noun to which it is attached and is transformed by adding an RC relativization marker, *d*e, instead of a relative pronoun in an English RC. The extracted object or subject falls in the clause-final position. Examples and structural representations of Chinese RCs are given in (2)^2^.

(2a)     Chinese SRC
              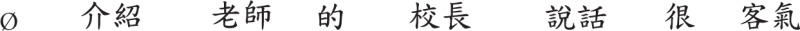              [*e*        jieshao       laoshi    de]_RC_    xiaozhang  shuohua    hen     keqi
              GAP   introduce    teacher  REL     principal     talk          very    polite
              “The principal who introduced the teacher talked in a very polite manner.”
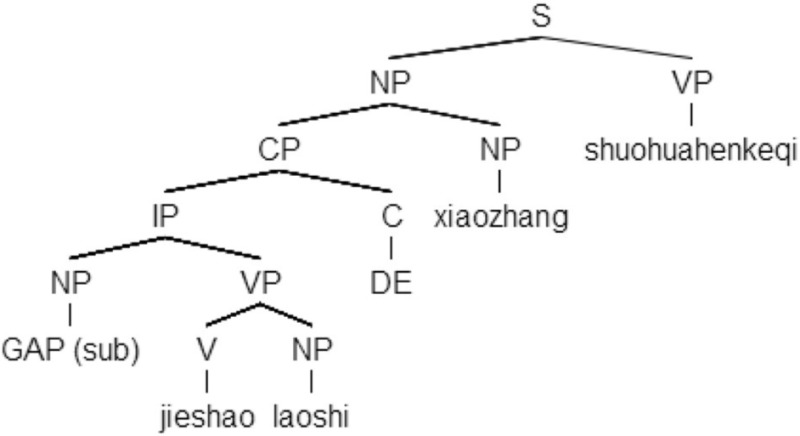
(2b)     Chinese ORC
              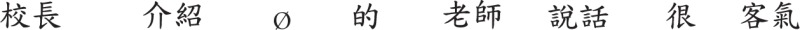               [xiaozhang  jieshao       *e*        de]_RC_    laoshi     shuohua   hen     keqi
               principal     introduce   GAP   REL      teacher   talk         very    polite
               “The teacher whom the principal introduced talked in a very polite manner.”
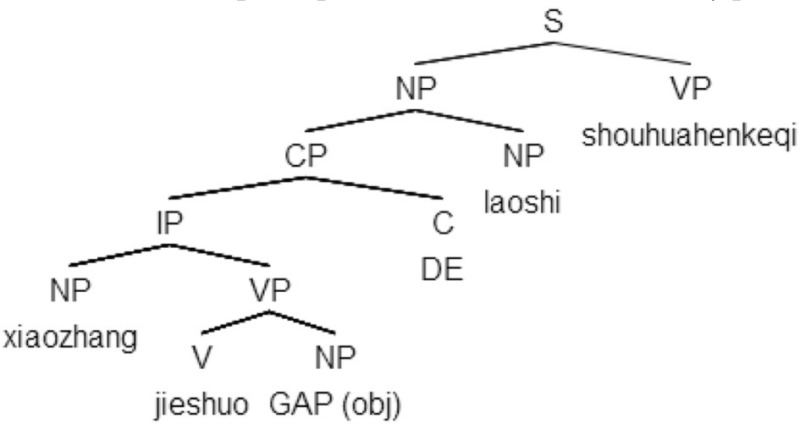

In (2a), the noun *xiaozhang* “principal,” extracted from the subject position of the embedded verb *jieshao* “introduce,” serves as the head noun of the RC introduced by the relativizer *de*. *De* is treated as a relativization marker and is considered as a complementizer in phrasal structure (Aoun and Li, 2003). The extracted noun *xiaozhang* is coindexed with the gap and fills that gap. In (2b), the noun *laoshi* “teacher” is extracted from the object position and therefore, forms an ORC. It is noted that Chinese RCs are prenominal, which means they precede head nouns.

The processing of Chinese RCs has been intensively investigated over the past few years. Finding both preference types in Chinese indicates that the universal SRC preference is not consistent across languages. Therefore, the presence of processing asymmetry in Chinese RCs raises the issues of whether the processing pattern of Chinese RCs is language-specific, and whether discordant findings are related to the syntactically mixed patterns in Chinese RCs.

The processing of Chinese RCs has been intensively investigated using different methods. Among these studies, SRC preference has been reported in self-paced reading tasks (e.g., Lin and Bever, 2006a,b, 2007, 2011), in computational modeling (Chen et al., 2012), and in the relative frequency of occurrence in the corpus (Vasishth et al., 2013). Among those studies supporting a universal SRC preference, Lin and Bever (2006a,b, 2007, 2011) demonstrated that SRCs are easier than ORCs in Chinese. They examined how readers process RCs from two perspectives: RC modification (subject-modifying RC vs. object-modifying RC) and RC embeddedness (singly embedded RCs vs. doubly embedded RCs). They found that the reading times on both the relativizer and the head noun were significantly shorter for SRCs than for ORCs, irrespective of whether the RC modifies the subject or the object of the main clause. Their results suggest an SRC preference in Chinese, which is in line with the findings across languages.

However, the finding that SRCs are easier to process in Chinese has been challenged by other reports of an ORC preference (e.g., in self-paced reading tasks, see Hsiao and Gibson, 2003; Hsu and Chen, 2007; Chen et al., 2008; Lin and Garnsey, 2011; Gibson and Wu, 2013; for a Mandarin-speaking aphasia case study, see Su et al., 2007). Among these works, the most often discussed is that of Hsiao and Gibson (2003). Those authors conducted a self-paced reading task with singly embedded and doubly embedded RCs, and with an RC-modifying subject of the main clauses. They found that in doubly embedded RCs, the reading times on the head noun and the embedded verb were shorter for ORCs than for SRCs. They demonstrated a preference for ORC in Chinese, implying that the processing of RCs in Chinese is language-specific.

#### Japanese RC structure and processing

Like Chinese RCs, Japanese RCs come before the head nouns they modify, and therefore Japanese exhibits a prenominal RC pattern. Examples of Japanese RCs are given in (3).

(3a)     Japanese SRC
             Ø           giin-          o          hinanshita        kisha
             [*e_*i*_*          senator-    ACC    attacked]_RC_    reporter
             GAP                                                         FILLER
             “the reporter (who) attacked the senator”
(3b)     Japanese ORC
             giin-         ga           Ø          hinanshita        kisha
             [senator   NOM      *e_*i*_*          attacked]_RC_    reporter
                                           GAP                            FILLER
             “the reporter (who) the senator attacked”
                                Adapted from Ueno and Garnsey (2008)

Japanese employs a different strategy to construct RCs, called the case-marking system, using case makers *o* and *ga* to indicate the syntactic function of the noun modified by the RC. Japanese does not have an overt relativizer, whereas Chinese has the relativizer *de* in RC.

Unlike reports of Chinese RCs having either an SRC and ORC preference, most research on the processing of Japanese RCs has demonstrated a preference for SRCs by native Japanese speakers (Miyamoto and Nakamura, 2003; Ueno and Garnsey, 2008).

#### Comparison of chinese and japanese structures

L2 sentence processing involves the syntactic structures of both L1 and L2. To understand and isolate the factors that potentially influence the processing of Chinese RCs by Japanese CSL learners, it is necessary to compare the structural properties of both involved languages. The structures of RC in Japanese and Chinese vary in several ways, as summarized in Table 1.

**Table 1:** **Comparison of syntactic properties between Japanese and Chinese**.

**Syntactic property**	**Chinese**	**Japanese**
Canonical word order	S-V-O	S-O-V
Head position	Head-initial	Head-final
RC position	Prenominal	Prenominal
Relativizer	Yes (RC marker: DE)	No (by case-making system)
Gap position	Before the HN	Before the HN

Due to the syntactic divergence between the two languages, it is expected that the different head positions between Chinese and Japanese may have certain effects on the L2 processing of Chinese, and therefore the two groups are hypothesized to show different processing patterns. Otherwise, if the two groups show similar processing patterns, then it would imply that the unique pattern of Chinese RCs may play a more crucial role.

### Theoretical accounts on L2 sentence processing

Regarding L2 sentence processing, three theoretical accounts have been proposed to explain the difference in processing patterns between SRCs and ORCs: Noun Phrase Accessibility Hierarchy (NPAH, Keenan and Comrie, 1977), Structural Distance Hypothesis (SDH), and Linear Distance Hypothesis (LDH).

#### Noun phrase accessibility hierarchy

Keenan and Comrie (1977) proposed a universal tendency, called the NPAH, which was derived from the observations of syntactic forms in a large number of languages. The NPAH ranks the accessibility of the syntactic positions in a sentence as follows: subject, direct object, indirect object, oblique object, possessor, and object of comparison. Accordingly, a language that can relativize a given position in the hierarchy can also relativize all of its antecedent positions. Some studies suggest that the difficulty processing RCs experienced by L2 learners is associated with the NPAH (Gass, 1979, 1982; Pavesi, 1986; Doughty, 1988, 1991; Eckman et al., 1988; Wolfe-Quintero, 1992; Izumi, 2003). The findings in those studies parallel the typological implications captured by the NPAH. The NPAH hypothesizes that the degree of accessibility to RC formation across languages, and such a universal tendency, implies that SRCs make sentence processing easier than ORCs.

#### Structural distance hypothesis

The SDH (Collins, 1994; Hamilton, 1995; O'Grady, 1997, 2001; O'Grady et al., 2003) states that the difficulty of an RC is determined by the depth of the gap corresponding to the relativized elements, and it is measured by counting the nodes between the gap and the filler of the RC.

In SRCs (e.g., 1a) the gap contains two phrasal nodes (IP and CP), while in ORCs (1b) the gap contains three phrasal nodes (VP, IP, and CP). Thus, the structural distance between the gap and its filler is greater in ORCs (1b) than in SRCs (1a).

#### Linear distance hypothesis

The LDH (Tarallo and Myhill, 1983; Hawkins, 1989; O'Grady, 2001) presents a more straightforward measurement for the gap-filler distance: counting the intervening elements (words or words with discourse referents) between the gap and filler. In SRCs (e.g., 1a), there are three words and one discourse referent intervening between the gap and its filler, and in ORCs (e.g., 1b) there is only one word along the same path. Hence, the linear distance between gap and filler is greater in SRCs (1a) than in ORCs (1b).

### L2 processing of RC

Regarding the processing of RCs by L2 learners, a great deal of previous studies have shown that L2 performance correlated with the prediction of NPAH (Gass, 1979, 1980, 1982; Hyltenstam, 1984; Pavesi, 1986; Doughty, 1988, 1991; Eckman et al., 1988; Wolfe-Quintero, 1992 among others). Those studies tested L2 learners with different L1 backgrounds through various tasks, including written sentence combination (Gass, 1979, 1980, 1982; Eckman et al., 1988), oral picture-cued production task (Pavesi, 1986), and guided oral production task (Wolfe-Quintero, 1992). The findings demonstrated that the participants performed better in SRCs than ORCs, implying that NPAH may still hold for the L2 sentence processing. Research on L2 processing in East Asian RCs, however, has arisen controversy to the acquisition difficulties across RC types (e.g., O'Grady et al., 2003; Jeon and Kim, 2007; Ozeki and Shirai, 2007; Yip and Matthews, 2007; Packard, 2008; Cui, 2013; Xu, 2014).

Several studies on the L2 processing of Chinese RC has reported inconsistent results with the acquisition difficulty/hierarchy made by NPAH. The results from those studies suggested that no clear processing asymmetry has been settled yet. First, Packard (2008) employed a self-paced reading task with both subject- and object-modifying SRCs and ORCs. He found that the participants read ORCs more quickly than SRCs. Moreover, Cui (2013) used a questionnaire and an online self-paced reading task to compare Chinese RC processing by L1 and L2 speakers. In her study, 24 native speakers of Chinese and 33 Chinese L2 learners (17 from head-initial L1 backgrounds and 16 from head-final L1 backgrounds) were recruited. The results of the questionnaire indicated that both L1 and L2 speakers found ORCs easier than SRCs. The data from the reading task showed that for L1 speakers, ORCs were read more quickly than SRCs only in subject-modifying RCs; for L2 speakers, no preference was found in the overall results, but an SRC preference was in the head-initial group. In addition, Xu (2014) conducted a written sentence combination task, testing 45 native English-speaking learners of Chinese on the production difficulty of four RC types: SRC, direct ORC, indirect ORC, object of preposition RC. She concluded that the participants' production difficulty fully follows the accessibility order of NPAH.

In a nutshell, the NPAH is typologically-driven and well-attested in many studies on L2 RC processing but it is still disputed for Chinese RCs. One may wonder if RC processing asymmetry is language- universal or specific in Chinese.

## The current study

This study focused on three major questions related to the conflicting research findings regarding processing of the two kinds of RCs:

1. Do speakers of Japanese (a head-final language) process Chinese RCs in a way similar to native speakers of Chinese (a head-initial language)?
2. How does the syntactic structure of L1 play a role in L2 processing of Chinese RCs?
3. Which of the theoretical hypotheses provides a better explanation of the results?

An eye-movement monitoring paradigm was employed to explore the reading patterns of Chinese RCs by Japanese CSL learners. The eye-movement technique enables us to obtain online information of consecutive reading as well as regression crucially relevant to RC processing. The eye-tracking indicators can be used to exam processing preference since fixation duration and the frequency of regressions increase as sentence becomes conceptually more difficult (Rayner, 1998).

### Interest areas and hypotheses

The interest areas, based on previous research, included the head noun and the embedded verb. These areas were examined in order to identify which type of RC is easier to process and where the processing difficulties (if any) arise. The head noun is of interest because it is the element that is extracted from the clause that later is transformed into an RC by adding the RC marker DE. In addition, head noun contrasts in the two RC types in terms of the syntactic functions (subject vs. object). The embedded verb was examined for two reasons: (1) the embedded verb syntactically governs the head noun of the RC, and (2) since Japanese is a head-final language, readers may pay special attention to the embedded verb, which may lead to different reading patterns from those of Chinese speakers.

The predictions of different theories regarding RCs, the head noun, and the embedded verb vary according to the factors emphasized, as follows:

1. The NPAH would predict an SRC preference because the subject is universally easier to relativize than the object, and participants would spend less time on the head noun of an SRC than of an ORC. That is, the processing time for an head noun would be *shorter* for an SRC than for an ORC. Although in the current study, all RCs modify the subjects of main clauses (subject-modifying RCs), within the RC structure, we compared the processing difference between SRCs and ORCs. Then, NPAH would favor SRCs. The NPAH assumption would lead to another prediction: that L1 syntax does not influence L2 sentence processing.
2. The SDH would predict an SRC preference in Chinese in that the structural distance between gap and filler (i.e., the depth of embeddedness of the gap) is shorter in a SRC than in a. ORC.
3. The LDH would predict an ORC preference in that the linear distance between the gap and its filler is shorter in a Chinese ORC than in a Chinese SRC.

The NPAH and SDH make the same prediction in Chinese RCs in terms of processing asymmetry. However, it should be noted that two accounts were based on different theoretical implications, the former came from observations of typologically different languages while the latter was built upon syntactic structures.

## Method

### Participants

Thirty-six native Japanese speakers were recruited at the Mandarin Training Center at National Taiwan Normal University. These participants had learned Chinese for a mean of 2.1 years, and their level of proficiency in the language corresponded to A1-B1 level according to their learning materials associated with the standard of the Common European Framework of Reference for Languages (CEFR). They came from classes of the same proficiency level. Thirty-eight native speakers of Mandarin Chinese who were college students were also recruited. The data of participants with a comprehension accuracy of <70% in the reading comprehension test were removed from the analysis. Based on the inclusion criteria, 33 native Japanese speakers and 38 native Chinese speakers were considered to be valid samples (11 male and 22 female Japanese, and 7 male, and 31 female Chinese). The native Japanese and Chinese speakers ranged in age from 21 to 50 years (*M* = 28.76 years) and 20 to 49 years (*M* = 22.42 years), respectively. All had normal or corrected-to-normal vision.

### Apparatus

The sentences were presented in black against a light-gray background on a 19-inch CHIMEI CMV A902 LCD monitor (1024 × 768-pixel resolution). Eye movements were recorded with an EyeLink 1000 eye tracker (SR Research, Canada). The sampling rate was set to 1000 Hz. The equipment comprised two personal computers (PCs) with Intel Core i5 3.2-GHz processors: one was a display PC that was responsible for presenting stimuli and controlling the experiment, and the other was a host PC that was responsible for monitoring and collecting eye-movement data. Participants were instructed to rest their head on a chinrest to minimize head movements. Although, viewing was performed binocularly, only data for the left eye were recorded. The programming was conducted using Experiment Builder 1.10.1 (SR Research Ltd. 2004–2010), and data were analyzed using Data Viewer 1.11.1 (SR Research Ltd. 2002–2011) and SPSS 18.

### Materials and design

The experiment had a 2 × 2 within-subject design. The independent variables were clause type (SRC vs. ORC) and distance (long vs. short), where distance was defined as the length between the gap and the head noun with which it was associated (long distance: 6–10 characters; short distance: 1–5 characters). The distance between the two syntactic dependents was manipulated by the additional modifiers preceding the head noun. The dependent variables were the accuracy rate in the reading comprehension test, reading time, gaze duration, regression-path duration, and regression rate.

The eye-movement task comprised 120 sentences: 60 experimental sentences (sentences with RCs) and 60 fillers (sentences without RCs). The experimental sentences comprised 30 SRCs and 30 ORCs, with 15 long-distance and 15 short-distance sentences in each. The frequencies of words in the experimental sentences were calculated based on the frequency list of 8000 Chinese words compiled by the Steering Committee for the Test of Proficiency (SC-TOP) in Huayu, Taiwan, to ensure that (a) reading could be performed without vocabulary difficulties and (b) all the experimental sentences comprised phrases with similar difficulties for L2 learners, with only the complexities in the RCs varying. The average frequency of head nouns in SRCs was 0.014 (*SD* = 0.015) and that in ORCs was 0.019 (*SD* = 0.029). The difference between average frequencies of head nouns in SRCs and ORCs was not significant [*t*_(14)_ = 1.03, *p* = 0.32]. The words used in the experimental sentences included the vocabulary at Level 1, 2, and 3 (corresponding to A1, A2, and B1 under CEFR) in Chinese 8000 words (SC-TOP). Sample stimuli are given in (4), where the head nouns and embedded verbs are bolded.

(4a)     Short-distance SRC
                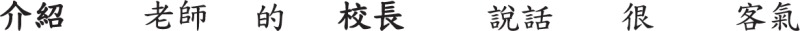                **jieshao**      laoshi     de      **xiaozhang**  shouhua    hen         keqi
                introduce    teacher   REL  principal      talk          very        politely
                “The principal who introduced the teacher talked in a very polite manner.”
(4b)     Short-distance ORC
                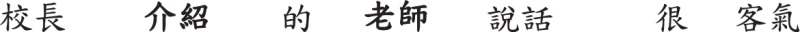                xiaozhang   **jieshao**     de       **laoshi**     shouhua        hen     keqi
                principal     introduce   REL    teacher    talk              very    politely
                “The teacher who the principal introduced talked in a very polite manner.”
(4c)      Long-distance SRC
                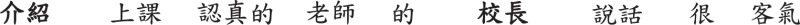                 **jieshao**    shangke-renzhen-de  laoshi    de          **xiaozhang**  shouhua    hen    keqi
                 introduce  seriously-teaching     teacher   REL      principal      talk          very   politely
                 “The principal who introduced the hard-working teacher talked in a very polite manner.”
(4d)      Long-distance ORC
                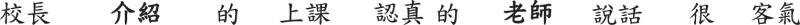                xiaozhang   **jieshao**     de      shangke-renzhen-de      **laoshi**    shouhua   hen     keqi
                principal     introduce   REL   seriously-teaching         teacher   talk          very    politely
                “The hard-working teacher who the principal introduced talked in a very polite manner.”

All experimental sentences and fillers were displayed in a single line in the middle of an LCD screen. The lengths of the sentences ranged from 12 to 21 characters, and the size of the characters was 36 × 36 pixels, with intercharacter spaces of 10 × 36 pixels. Each of the sentences was presented horizontally from left to right on the screen. The fillers covered various sentence structures, such as ba-construction and bei-construction. The fillers varied in length since the critical sentences included long-distance and short-distance sentences.

### Data analysis

The data analysis included the accuracy of the reading comprehension test, reading time of full sentences, and eye-movement data. The eye-movement data were measured according to the gaze duration, regression-path duration, and regression rate. The gaze duration is the sum of all first-pass fixations on a region before the eyes move out of the region to either the right or left (Rayner, 1998). The regression-path duration is the total time spent fixating on all of the target and pretarget regions, from the first fixation on a target region to fixation to the right of the target region (Rayner and Duffy, 1986; Liversedge et al., 1998). The regression rate is corresponding to the probability of rereading the target (Yen et al., 2008), i.e., the probability of regressions back into the target region after it has already been read. Fixations of <80 or >1200 ms (representing 4.15 and 5.00% of Japanese and Chinese language groups, respectively) were eliminated from the analyses (Liversedge et al., 2004; Drieghe et al., 2008; White, 2008; Slattery et al., 2013; Stites et al., 2013). Two-way repeated-measures ANOVAs were conducted for participants (*F*_1_) and items (*F*_2_). The analysis of eye-movement data was based on the trials in which no errors were made in the reading comprehension test.

### Procedure

This study was approved by National Science council in Taiwan with written informed consent from all participants. The participants were asked to provide background information before they proceeded to begin doing the experiment. The eye-movement task, which took about 25 min for L1 participants and 40 min for L2 participants to complete, was then conducted. Each participant sat 70 cm in front of a screen, with his or her head leaning on a chinrest. At this viewing distance, each character subtended a visual angle of 1.06°. The task began with a 13-point calibration, followed by five practice trials. The practice trials were in the same format as the experimental trials. For drift correction, the participants were instructed to look at a dot positioned at the location of the first character of the sentence. After the participants fixated on the dot, the experimenter pressed a button and the sentence appeared on the screen. The participants viewed each complete sentence on the screen one at a time, and were instructed to read each sentence at their own pace. Their eye movements were tracked while reading the sentence. When the participants were finished reading, they were asked to answer a true-or-false reading comprehension question to ensure that they had understood the sentence. Once the reading comprehension question was presented, the subjects were unable to go back to the test sentence. There were 120 questions in total, with a 5-min break every 40 trials. The 13-point calibration was readministered after each break. The sentences were presented in a randomized order, meaning that each participant viewed them in a different order.

## Results

### Accuracy of the reading comprehension test

The means and standard deviations for accuracy of the comprehension test are given in Table 2.

**Table 2:** **Mean (*M*) and standard deviation (*SD*) values for the accuracy of the post-stimulus reading comprehension test**.

**Type**	**Chinese**				**Japanese**			
	**Long**		**Short**		**Long**		**Short**	
	***M***	***SD***	***M***	***SD***	***M***	***SD***	***M***	***SD***
ORC	0.970	0.057	0.968	0.055	0.889	0.093	0.884	0.153
SRC	0.977	0.053	0.982	0.056	0.865	0.169	0.925	0.087

*Long and short refer to the distance between the gap and the HN*.

For native Chinese speakers, the main effect of clause type on accuracy was marginally significant [*F*_1(1, 37)_ = 3.91, *MSE* = 0.001, *p* = 0.055, partial eta squared (η^2^) = 0.10; *F*_2(1, 14)_ = 0.96, *MSE* = 0.002, *p* = 0.34, η^2^ = 0.08]. The accuracy was higher for SRCs (*M* = 0.980, *SD* = 0.055) than for ORCs (*M* = 0.969, *SD* = 0.056). However, there was no significant main effect of distance or significant interaction of clause type and distance (all *ps* > 0.50).

For Japanese speakers, the main effect of distance on accuracy was significant in the analysis by participants [*F*_1(1, 32)_ = 4.15, *MSE* = 0.006, *p* < 0.05, η^2^ = 0.11; *F*_2(1, 14)_ = 0.05, *MSE* = 0.019, *p* = 0.83, η^2^ = 0.00]. The accuracy was higher for short-distance sentences (*M* = 0.905, *SD* = 0.126) than for long-distance sentences (*M* = 0.877, *SD* = 0.137). However, there was no significant main effect of clause type or significant interaction of clause type and distance (all *p* > 0.10).

### Reading time of full sentences

The means and standard deviations for reading time of full sentences are given in Table 3.

**Table 3:** **Mean (M) and standard deviation (SD) values for Reading time of full sentences (sec)**.

**Type**	**Chinese**				**Japanese**			
	**Long**		**Short**		**Long**		**Short**	
	***M***	***SD***	***M***	***SD***	***M***	***SD***	***M***	***SD***
ORC	4.37	1.30	3.10	0.90	8.13	1.41	5.59	1.03
SRC	4.98	1.24	3.13	0.68	9.14	1.81	6.13	1.15

*Long and short refer to the distance between the gap and the HN*.

**Figure 1:**
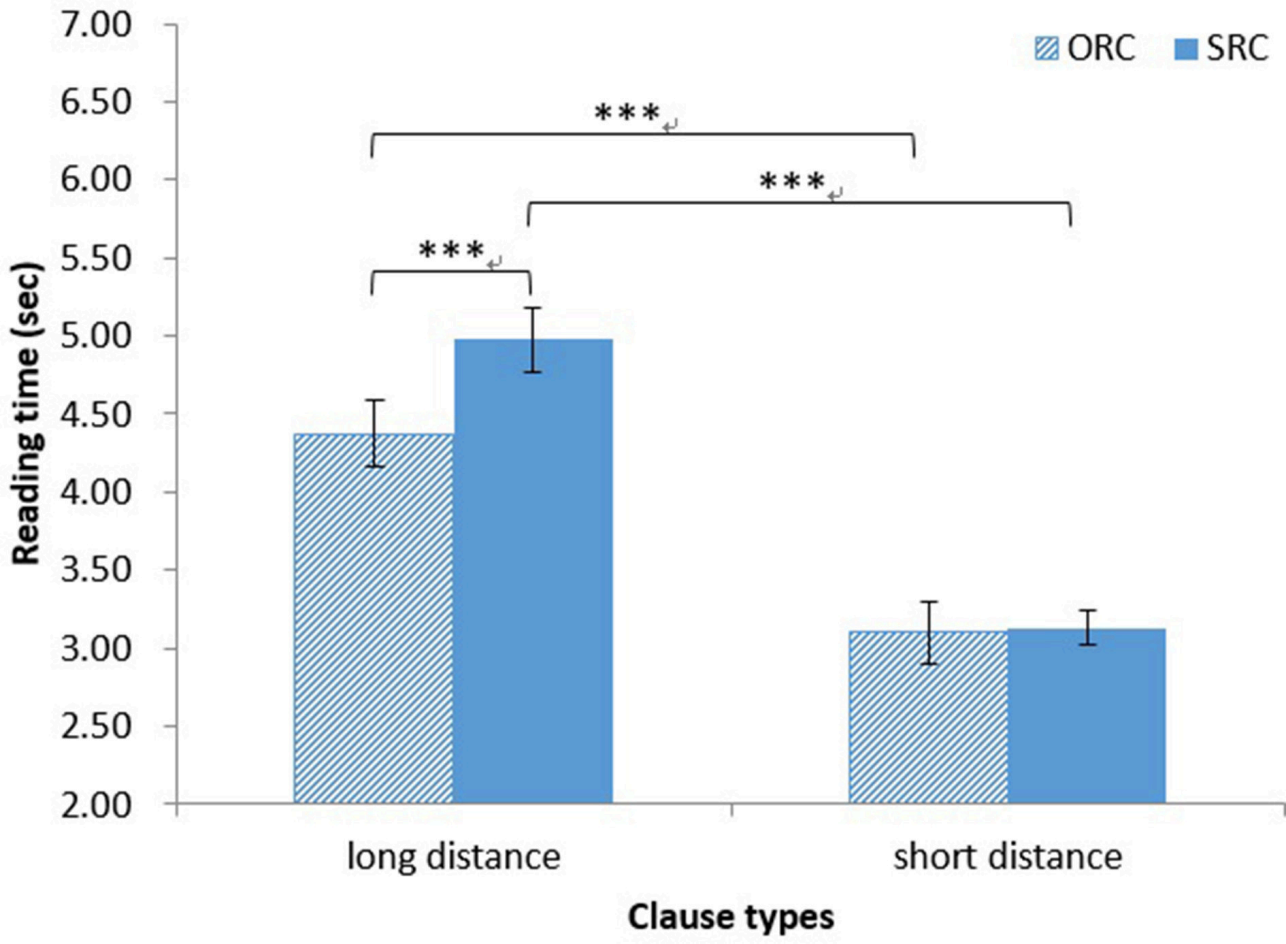
**Chinese group: reading time of full sentences**. ^***^p < 0.001.

For native Chinese speakers, the main effect of clause type on reading time was significant [*F*_1(1, 37)_ = 16.50, *MSE* = 0.233, *p* < 0.001, η^2^ = 0.31; *F*_2(1, 14)_ = 11.61, *MSE* = 0.136, *p* < 0.01, η^2^ = 0.45]. The reading time was significantly longer for full sentences in SRCs (*M* = 4.05 s, *SD* = 1.36 s) than for those in ORCs (*M* = 3.74 s, *SD* = 1.29 s). The main effect of distance on reading time was significant [*F*_1(1, 37)_ = 181.89, *MSE* = 0.507, *p* < 0.001, η^2^ = 0.83; *F*_2(1, 14)_ = 449.28, *MSE* = 0.077, *p* < 0.001, η^2^ = 0.97]. The reading time was significantly longer for long-distance sentences (*M* = 4.67 s, *SD* = 1.31 s) than for short-distance sentences (*M* = 3.12 s, *SD* = 0.80 s). The interaction between clause type and distance was also significant [*F*_1(1, 37)_ = 10.41, *MSE* = 0.298, *p* < 0.01, η^2^ = 0.22; *F*_2(1, 14)_ = 12.00, *MSE* = 0.120, *p* < 0.01, η^2^ = 0.46]. A simple main effect test showed that the reading time was significantly longer for full sentences in long-distance SRCs (*M* = 4.94 ms, *SD* = 0.64 s) than for those in long-distance ORCs (*M* = 4.31 s, *SD* = 0.33 ms) [*F*_1_(1, 74) = 26.08, *MSE* = 0.265, *p* < 0.001, η^2^ = 0.26; *F*_2(1, 28)_ = 23.58, *MSE* = 0.128, *p* < 0.001, η^2^ = 0.46]. Regardless of clause type, the reading time was significantly longer for long-distance sentences than for short-distance sentences (all *ps* < 0.001; see Figure 1).

For Japanese speakers, the main effect of clause type on reading time was significant [*F*_1(1, 32)_ = 41.54, *MSE* = 0.481, *p* < 0.001, η^2^ = 0.56; *F*_2(1, 14)_ = 24.59, *MSE* = 0.399, *p* < 0.001, η^2^ = 0.64]. The reading time was significantly longer for sentences in SRCs (*M* = 7.64 s, *SD* = 2.13 s) than for those in ORCs (*M* = 6.86 s, *SD* = 1.77 s). The main effect of distance on reading time was significant [*F*_1(1, 32)_ = 203.67, *MSE* = 1.244, *p* < 0.001, η^2^ = 0.86; *F*_2(1, 14)_ = 324.52, *MSE* = 0.370, *p* < 0.001, η^2^ = 0.96]. The reading time was significantly longer for long-distance sentences (*M* = 8.63 s, *SD* = 1.70 s) than for short-distance sentences (*M* = 5.86 s, *SD* = 1.13 s). However, there was no significant interaction of clause type and distance (both *ps* > 0.06).

### Eye-movement data

Table 4 lists the descriptive statistics for the gaze duration, regression-path duration, and regression rate. These indices are discussed in detail below.

**Table 4:** **Data for eye-movement indices for each critical region**.

	**Chinese**				**Japanese**			
	**HN**		**EV**		**HN**		**EV**	
	***M***	***SD***	***M***	***SD***	***M***	***SD***	***M***	***SD***
**GAZE DURATION (ms)**								
ORC-L	215	26	277	51	341	65	345	67
ORC-S	224	34	272	51	371	76	346	78
SRC-L	243	39	241	57	370	70	414	139
SRC-S	233	42	231	52	364	83	397	123
**REGRESSION-PATH DURATION (ms)**								
ORC-L	294	72	N/A		434	133	N/A	
ORC-S	387	105	N/A		512	160	N/A	
SRC-L	535	184	N/A		642	293	N/A	
SRC-S	472	134	N/A		564	197	N/A	
**REGRESSION RATE**								
ORC-L	0.32	0.15	0.66	0.21	0.39	0.17	0.69	0.20
ORC-S	0.35	0.19	0.70	0.18	0.43	0.17	0.71	0.18
SRC-L	0.28	0.17	0.70	0.28	0.38	0.18	0.78	0.17
SRC-S	0.32	0.15	0.62	0.30	0.36	0.18	0.78	0.19

*L, long distance between the gap and the HN; S, short distance between the gap and the HN; N/A, not acquired*.

#### Chinese speakers

##### Head nouns

Head nouns were measured using gaze duration, regression-path duration, and regression rate.

**Figure 2:**
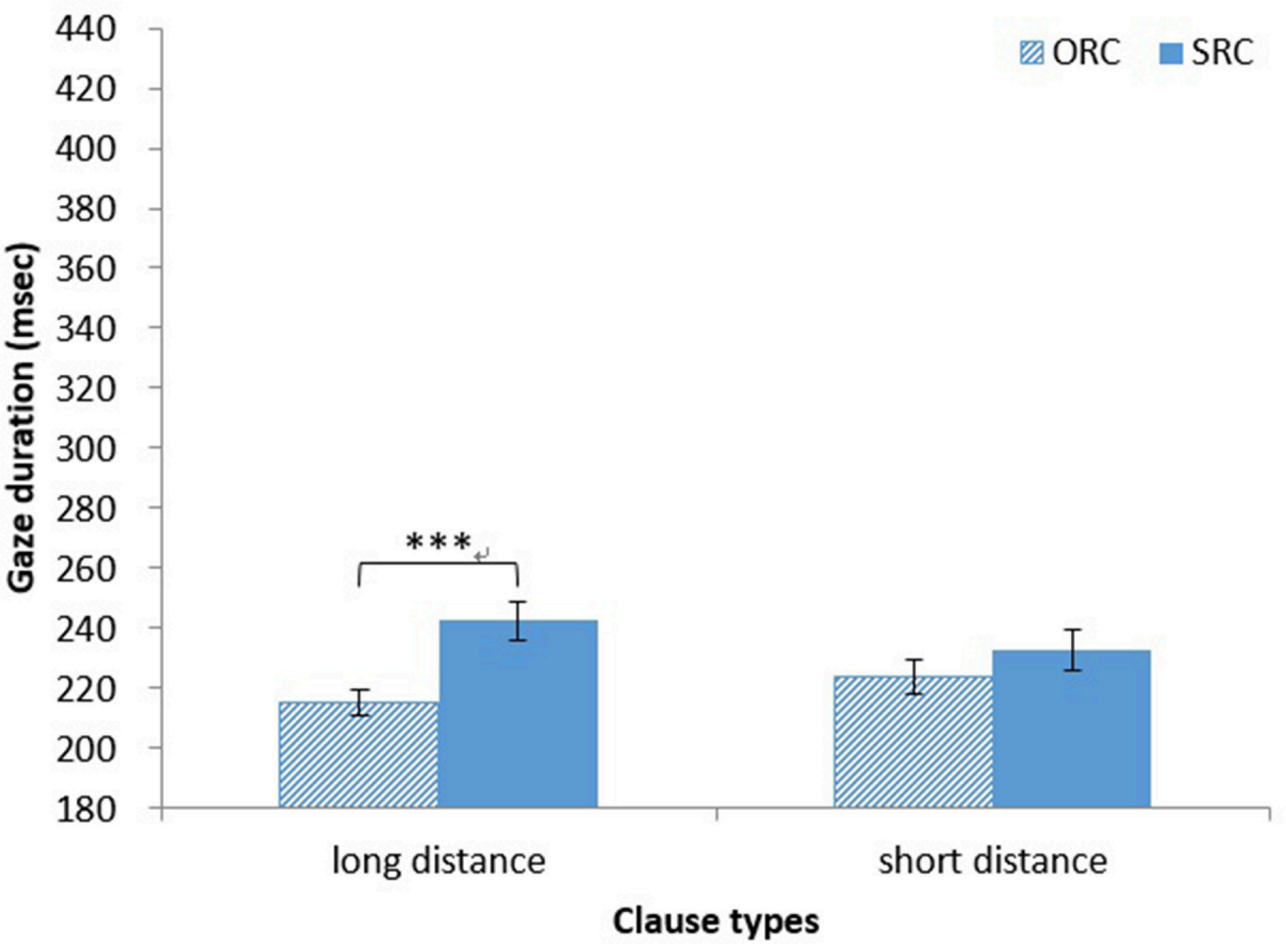
**Chinese group: gaze duration for HNs in RCs**. ^***^p < 0.001.

*Gaze duration*. The main effect of clause type on gaze duration was significant [*F*_1(1, 37)_ = 16.75, *MSE* = 744, *p* < 0.001, η^2^ = 0.31; *F*_2(1, 14)_ = 12.08, *MSE* = 390, *p* < 0.01, η^2^ = 0.46]. The gaze duration was significantly longer for head nouns in SRCs (*M* = 238 ms, *SD* = 41 ms) than for those in ORCs (*M* = 220 ms, *SD* = 31 ms). The main effect of distance was not significant (both *ps* > 0.90). The interaction between clause type and distance was also significant in the analysis by participants [*F*_1(1, 37)_ = 5.64, *MSE* = 570, *p* < 0.05, η^2^ = 0.13; *F*_2(1, 14)_ = 3.59, *MSE* = 306, *p* = 0.08, η^2^ = 0.20]. A simple main effect test showed that the gaze duration was significantly longer for Head nouns in long-distance SRCs (*M* = 243 ms, *SD* = 39 ms) than for those in long-distance ORCs (*M* = 215 ms, *SD* = 26 ms) [*F*_1(1, 74)_ = 21.56, *MSE* = 657, *p* < 0.001, η^2^ = 0.23; *F*_2(1, 28)_ = 14.88, *MSE* = 348, *p* < 0.001, η^2^ = 0.35; see Figure 2].

**Figure 3:**
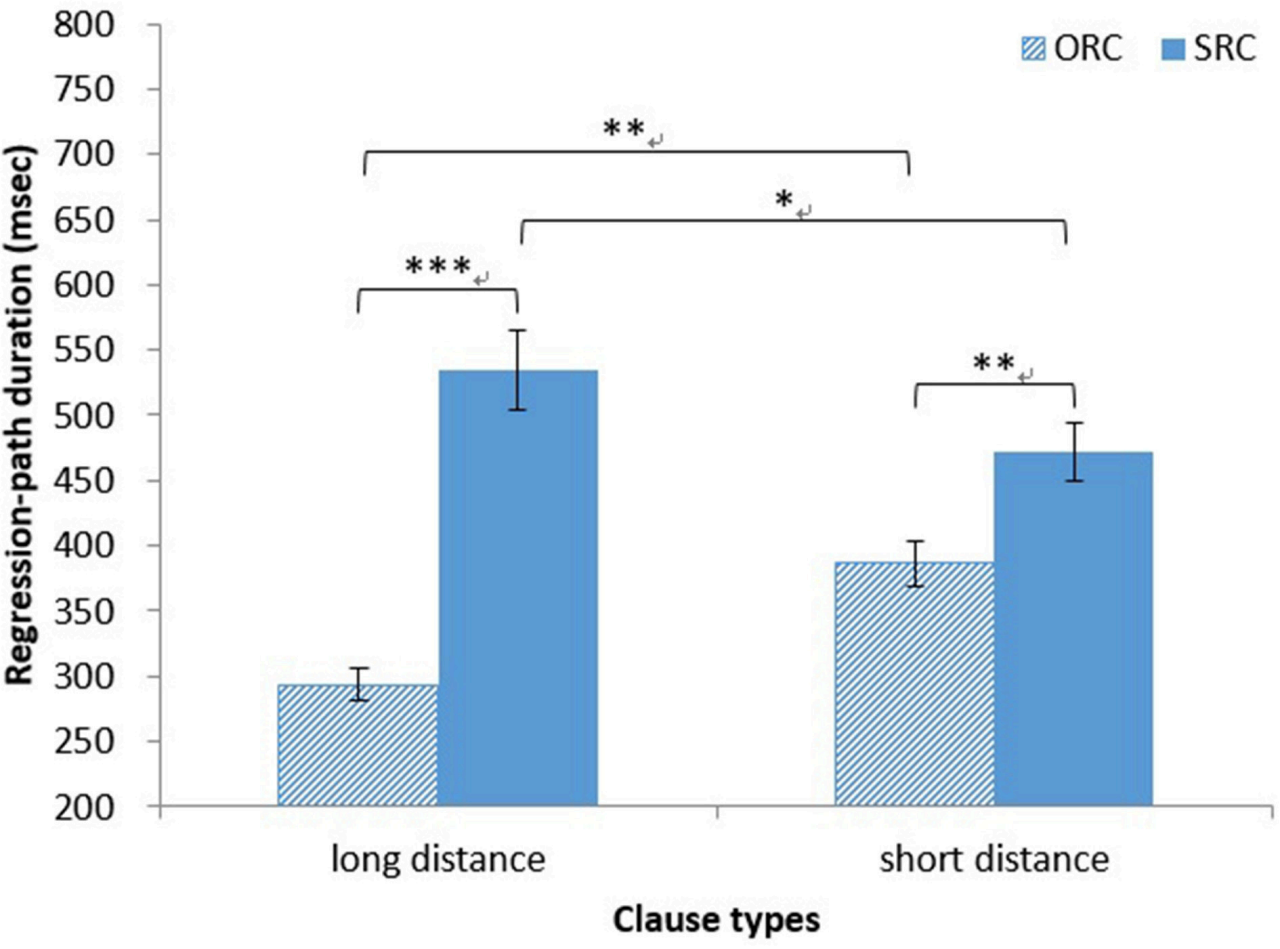
**Chinese group: regression-path duration for HNs in RCs**. ^*^p < 0.05, ^**^p < 0.01, and ^***^p < 0.001.

*Regression-path duration*. The main effect of clause type on regression-path duration was significant [*F*_1(1, 37)_ = 69.01, *MSE* = 14, 662, *p* < 0.001, η^2^ = 0.65; *F*_2(1, 14)_ = 56.97, *MSE* = 6828, *p* < 0.001, η^2^ = 0.80]. The regression-path duration was significantly longer for head nouns in SRCs (*M* = 503 ms, *SD* = 164 ms) than for those in ORCs (*M* = 340 ms, *SD* = 101 ms). The main effect of distance was not significant (both *ps* > 0.30). The interaction between clause type and distance was also significant [*F*_1(1, 37)_ = 21.85, *MSE* = 10, 461, *p* < 0.001, η^2^ = 0.37; *F*_2(1, 14)_ = 38.42, *MSE* = 2629, *p* < 0.001, η^2^ = 0.73]. A simple main effect test showed that the regression-path duration was significantly longer for head nouns in long-distance SRCs (*M* = 535 ms, *SD* = 184 ms) than for those in long-distance ORCs (*M* = 294 ms, *SD* = 72 ms) [*F*_1(1, 74)_ = 87.66, *MSE* = 12, 561, *p* < 0.001, η^2^ = 0.54; *F*_2(1, 28)_ = 93.73, *MSE* = 4728, *p* < 0.001, η^2^ = 0.77]. The regression-path duration was significantly longer for head nouns in short-distance SRCs (*M* = 472 ms, *SD* = 134 ms) than for those in short-distance ORCs (*M* = 387 ms, *SD* = 105 ms) [*F*_1(1, 74)_ = 11.09, *MSE* = 12, 561, *p* < 0.01, η^2^ = 0.13; *F*_2(1, 28)_ = 9.90, *MSE* = 4728, *p* < 0.01, η^2^ = 0.26]. The regression-path duration was significantly longer for head nouns in long-distance SRCs (*M* = 535 ms, *SD* = 184 ms) than for those in short-distance SRCs by items (*M* = 472 ms, *SD* = 134 ms) [*F*_1_(1, 74) = 5.15, *MSE* = 14, 339, *p* = 0.03, η^2^ = 0.07; *F*_2(1, 28)_ = 10.35, *MSE* = 3328, *p* < 0.01, η^2^ = 0.27]. The regression-path duration was significantly longer for head nouns in short-distance ORCs (*M* = 387 ms, *SD* = 105 ms) than for those in long-distance ORCs (*M* = 294 ms, *SD* = 72 ms) [*F*_1(1, 74)_ = 11.41, *MSE* = 14, 339, *p* < 0.01, η^2^ = 0.13; *F*_2(1, 28)_ = 20.92, *MSE* = 3328, *p* < 0.001, η^2^ = 0.43] Regardless of the sentence distance, the regression-path duration was significantly longer for head nouns in SRCs than for those in ORCs (all *ps* < 0.01; see Figure 3).

*Regression rate*. The main effect of clause type on regression rate was not significant (both *ps* > 0.10), as were the main effect of distance (both *ps* > 0.05) and the interaction between clause type and distance (both *ps* > 0.80).

The results for head nouns showed that gaze duration and regression-path duration on SRCs were longer than those on ORCs. Hence, the results generally suggested an ORC preference for Chinese speakers.

##### Embedded verbs

Embedded verbs were measured using gaze duration and regression rate. Note that the measure of regression-path duration was not used since the positions of the embedded verbs in the two types of RCs are different. In particular, the embedded verb of SRC is in sentence-initial position so that its regression time may be underestimated. Thus, the indicator regression-path duration involving regression time was excluded for the analysis of embedded verbs.

*Gaze duration*. The main effect of clause type on gaze duration was significant [*F*_1(1, 37)_ = 19.98, *MSE* = 2819, *p* < 0.001, η^2^ = 0.35; *F*_2(1, 14)_ = 84.35, *MSE* = 202, *p* < 0.001, η^2^ = 0.86]. The gaze duration was significantly longer for embedded verbs in ORCs (*M* = 274 ms, *SD* = 51 ms) than for those in SRCs (*M* = 236 ms, *SD* = 55 ms). However, there was no significant main effect of distance or significant interaction of clause type and distance (all *ps* > 0.10).

**Figure 4:**
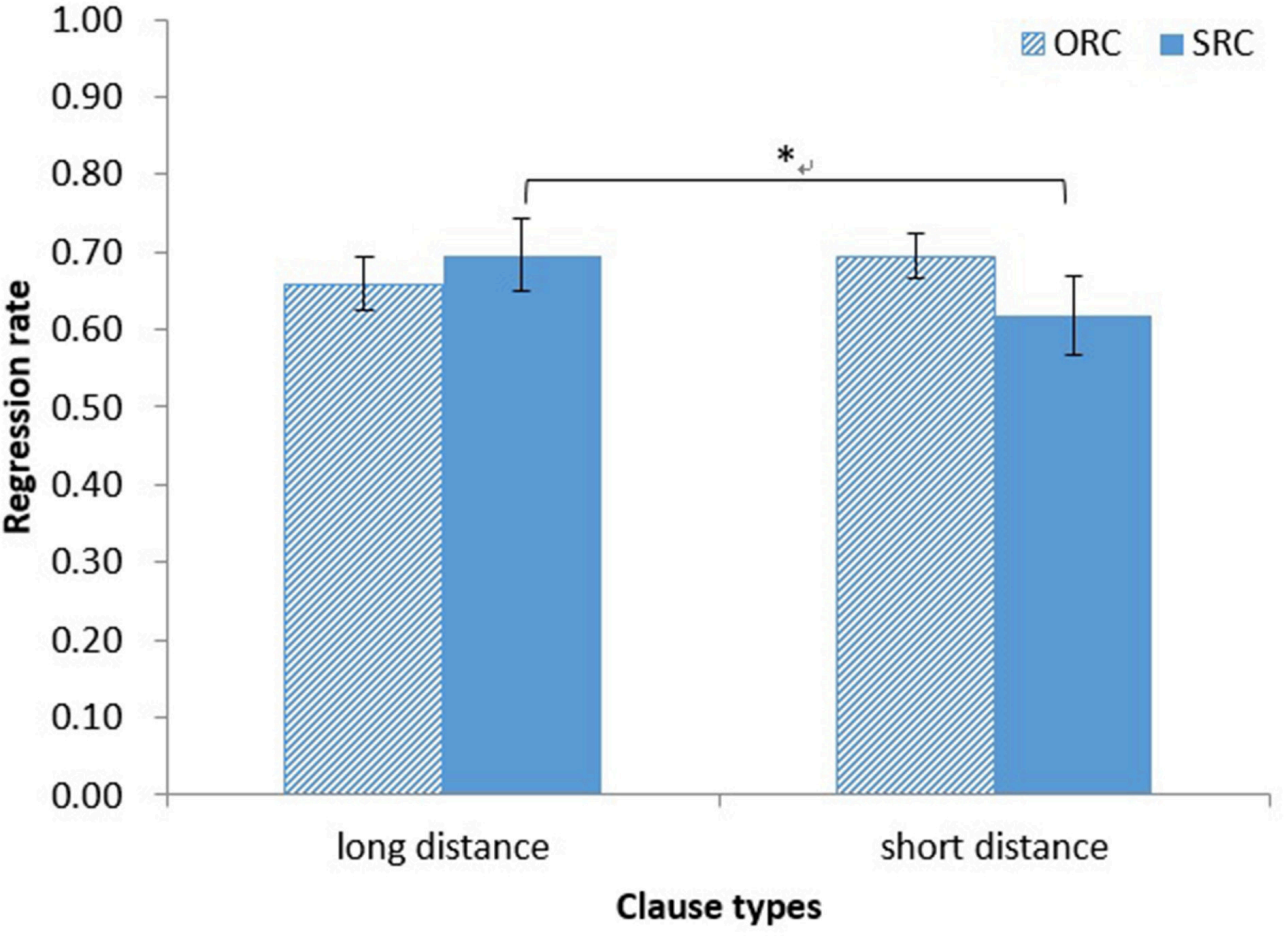
**Chinese group: regression rate for EVs in RCs**. ^*^p < 0.05.

*Regression rate*. The main effect of clause type on regression rate was not significant (both *ps* > 0.10), as was the main effect of distance (both *ps* > 0.30). However, the interaction between clause type and distance was significant [*F*_1(1, 37)_ = 6.75, *MSE* = 0.018, *p* < 0.05, η^2^ = 0.15; *F*_2(1, 14)_ = 4.59, *MSE* = 0.005, *p* = 0.05, η^2^ = 0.25]. A simple main effect test showed that the regression rate was marginally significantly longer for embedded verbs in long-distance SRCs (*M* = 0.70, *SD* = 0.28) than for those in short-distance SRCs in the analysis by participants (*M* = 0.62, *SD* = 0.30) [*F*_1(1, 74)_ = 5.54, *MSE* = 0.021, *p* = 0.02, η^2^ = 0.07; *F*_2(1, 28)_ = 1.03, *MSE* = 0.011, *p* = 0.32, η^2^ = 0.04; see Figure 4].

The results for embedded verbs showed that gaze duration on ORCs was longer than that on SRCs. The interpretation of such finding will be particularly discussed in Section ORC Processing Preference.

#### Japanese speakers

##### Head nouns

Head nouns were measured using gaze duration, regression-path duration, and regression rate.

**Figure 5:**
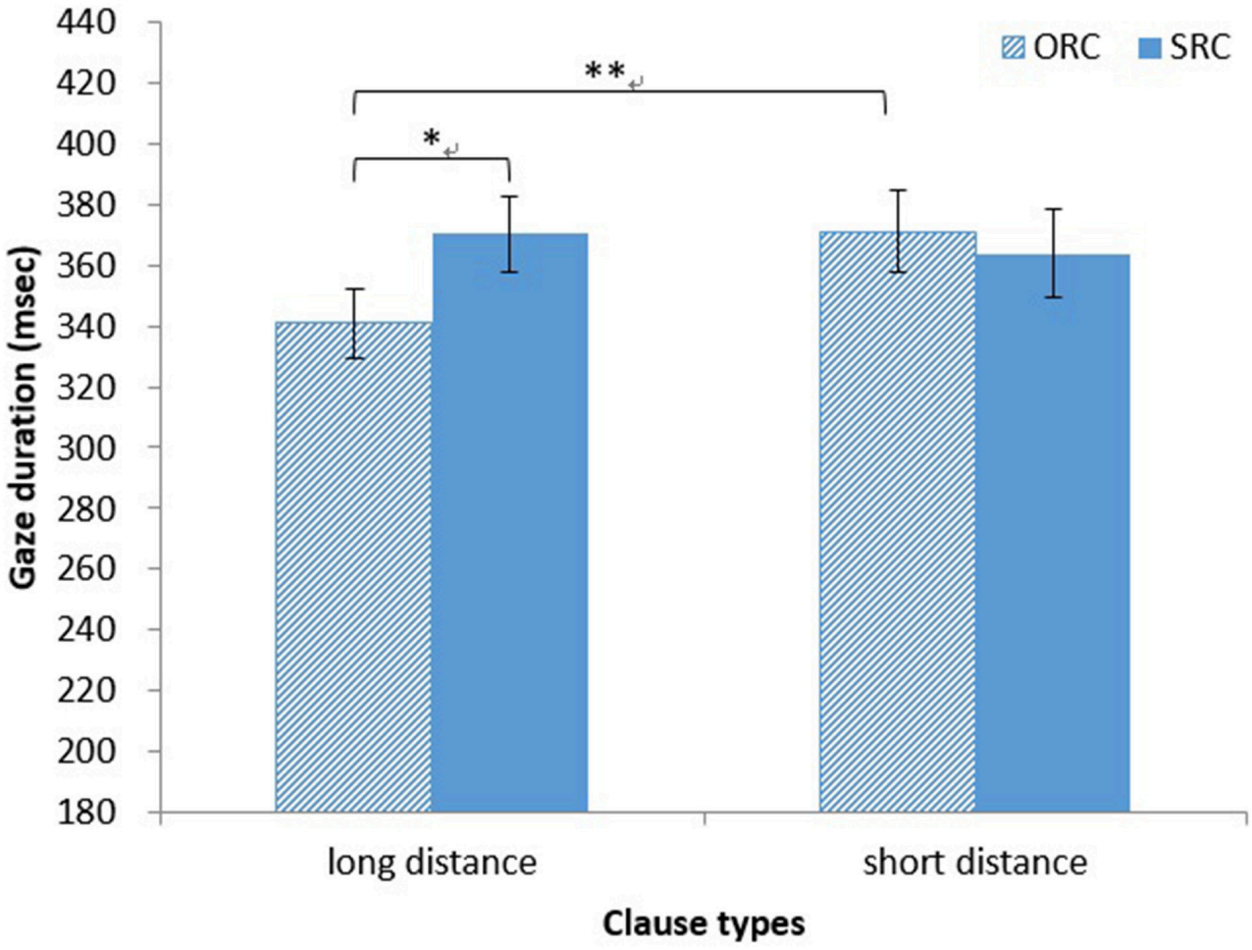
**Japanese group: gaze duration for HNs in RCs**. ^*^p < 0.05, ^**^p < 0.01.

*Gaze duration*. The main effect of clause type on gaze duration was not significant (both *ps* > 0.20), as was the main effect of distance (both *ps* > 0.05). However, the interaction between clause type and distance was significant [*F*_1(1, 32)_ = 4.56, *MSE* = 2393, *p* < 0.05, η^2^ = 0.13; *F*_2(1, 14)_ = 7.69, *MSE* = 605, *p* < 0.05, η^2^ = 0.35]. A simple main effect test showed that the gaze duration was significantly longer for head nouns in long-distance SRCs (*M* = 370 ms, *SD* = 70 ms) than for those in long-distance ORCs (*M* = 341 ms, *SD* = 65 ms) [*F*_1(1, 64)_ = 5.86, *MSE* = 2401, *p* < 0.05, η^2^ = 0.08; *F*_2(1, 28)_ = 5.29, *MSE* = 1516, *p* < 0.05, η^2^ = 0.16]. The gaze duration was significantly longer for head nouns in short-distance ORCs (*M* = 371 ms, *SD* = 76 ms) than for those in long-distance ORCs [*F*_1(1, 64)_ = 7.26, *MSE* = 2033, *p* < 0.01, η^2^ = 0.10; *F*_2(1, 28)_ = 10.94, *MSE* = 568, *p* < 0.01, η^2^ = 0.28; see Figure 5].

**Figure 6:**
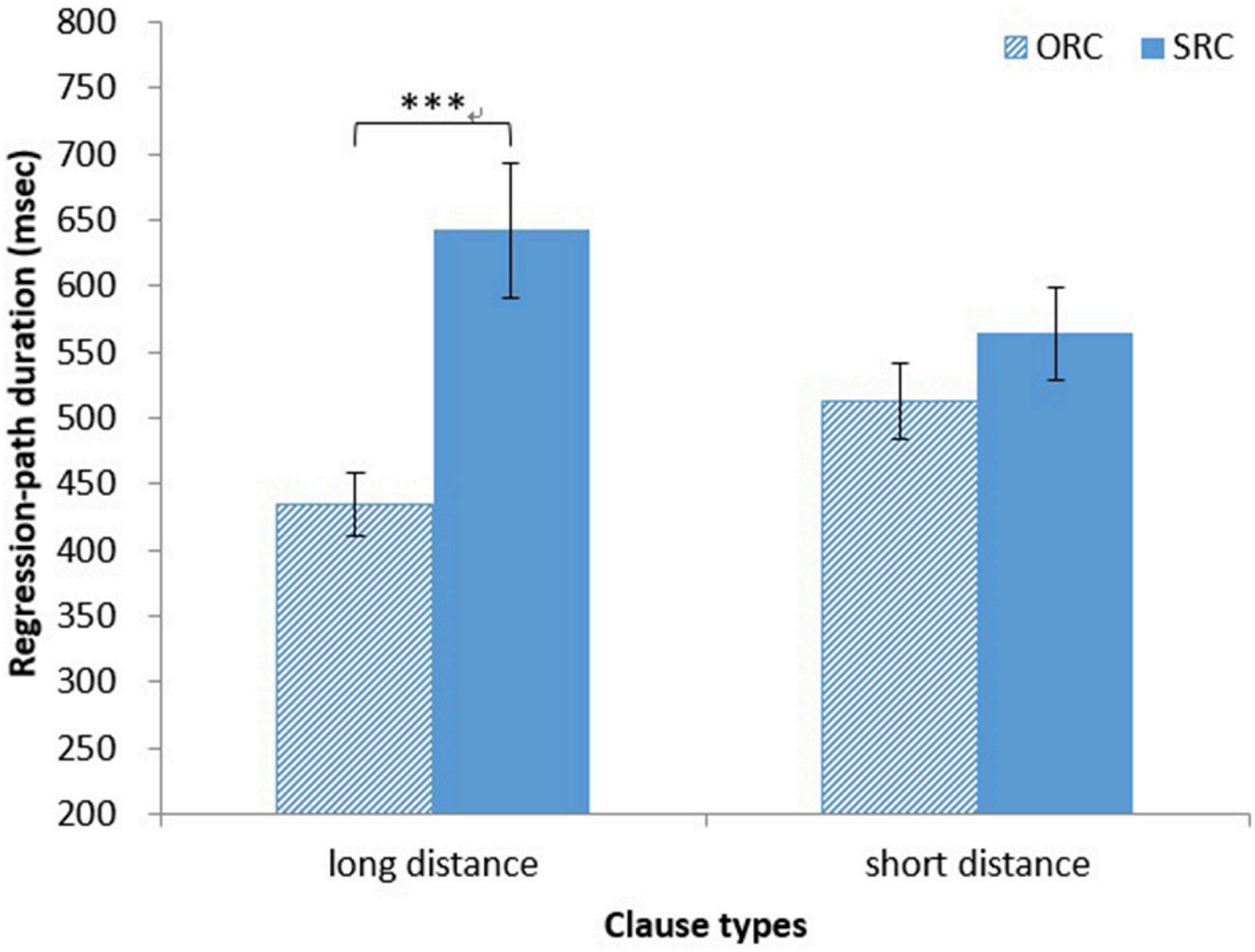
**Japanese group: regression-path durations for HNs in RCs**. ^***^p < 0.001.

*Regression-path duration*. The main effect of clause type on regression-path duration was significant [*F*_1(1, 32)_ = 20.14, *MSE* = 27, 567, *p* < 0.001, η^2^ = 0.39; *F*_2(1, 14)_ = 20.43, *MSE* = 14, 430, *p* < 0.001, η^2^ = 0.59]. The regression-path duration was significantly longer for head nouns in SRCs (*M* = 603 ms, *SD* = 253 ms) than for those in ORCs (*M* = 473 ms, *SD* = 152 ms). The main effect of distance was not significant (both *ps* > 0.90). The interaction between clause type and distance was also significant [*F*_1(1, 32)_ = 8.10, *MSE* = 24, 939, *p* < 0.01, η^2^ = 0.20; *F*_2(1, 14)_ = 12.80, *MSE* = 7446, *p* < 0.01, η^2^ = 0.48]. A simple main effect test showed that the regression-path duration was significantly longer for head nouns in long-distance SRCs (*M* = 642 ms, *SD* = 293 ms) than for those in long-distance ORCs (*M* = 434 ms, *SD* = 133 ms; [*F*_1(1, 64)_ = 27.17, *MSE* = 26, 253, *p* < 0.001, η^2^ = 0.30; *F*_2(1, 28)_ = 33.15, *MSE* = 10, 938, *p* < 0.001, η^2^ = 0.54; see Figure 6].

*Regression rate*. The main effect of clause type on regression rate was not significant (both *ps* > 0.05), as were the main effect of distance (both *ps* > 0.60) and the interaction between clause type and distance (both *ps* > 0.10).

The results for head nouns showed that gaze duration and regression-path duration on SRCs were longer than those on ORCs. The results generally suggested an ORC preference for Japanese speakers.

##### Embedded verbs

Embedded verbs were measured using gaze duration and regression rate.

*Gaze duration*. The main effect of clause type on gaze duration was significant [*F*_1(1, 32)_ = 8.23, *MSE* = 14, 339, *p* < 0.01, η^2^ = 0.20; *F*_2(1, 14)_ = 15.87, *MSE* = 2754, *p* < 0.01, η^2^ = 0.53]. The gaze duration was significantly longer for embedded verbs in SRCs (*M* = 406 ms, *SD* = 132 ms) than for those in ORCs (*M* = 346 ms, *SD* = 73 ms). However, there was no significant main effect of distance or significant interaction between clause type and distance (all *ps* > 0.30).

*Regression rate*. The main effect of clause type on regression rate was significant [*F*_1(1, 32)_ = 6.80, *MSE* = 0.033, *p* < 0.05, η^2^ = 0.18; *F*_2(1, 14)_ = 42.31, *MSE* = 0.002, *p* < 0.001, η^2^ = 0.75]. The regression rate was significantly higher for embedded verbs in SRCs (*M* = 0.78, *SD* = 0.18) than for those in ORCs (*M* = 0.70, *SD* = 0.19). However, there was no significant main effect of distance or significant interaction between clause type and distance (all *ps* > 0.20).

The results for embedded verbs showed that gaze duration on SRCs was longer than that on ORCs and regression rate for SRCs was higher than that for ORCs. The results generally suggested an ORC preference for Japanese speakers.

Taken together, the results comparing (1) SRC and ORC processing in the long-distance conditions and (2) SRC and ORC processing in the short-distance conditions are summarized in Table 5.

**Table 5:** **Summary Table: results of clause type, and clause type × distance**.

	**Chinese**		**Japanese**	
	**HN**	**EV**	**HN**	**EV**
Gaze duration	ORC < SRC ORC-L < SRC-L	SRC < ORC	ORC-L < SRC-L	ORC < SRC
Regression-path duration	ORC < SRC	N/A	ORC < SRC ORC-L < SRC-L	N/A
Regression rate	No significant difference	##	No significant difference	ORC < SRC

*The results comparing (1) short-distance and long-distance SRC processing and (2) short-distance and long-distance ORC processing are NOT presented here. The symbol ## indicates that the significant result here (SRC-L > SRC- S) is not relevant to the discussion on the interaction between clause type and distance*.

The overall results, as shown by the indicators in Table 4, suggested that the Japanese group revealed an ORC preference. Similarly, the Chinese group demonstrated an ORC preference, except for the gaze duration of embedded verbs, which will be discussed in next section.

## Discussion

In this study we used an eye-tracking technique to explore the difficulty experienced by Japanese learners when they are processing Chinese RCs. Overall, the results showed that ORCs were easier to process for Japanese CSL learners, which is similar to the pattern exhibited by Chinese speakers in terms of processing asymmetry. Our findings were consistent with the predictions of accounts based on the LDH, but not those of the NPAH and SDH. In this section we discuss the findings under the theoretical framework of RC processing in order to address our research questions, and then consider the implications for L2 sentence comprehension.

### ORC processing preference

The results generally suggest an ORC preference for both the Chinese speakers and Japanese CSL learners. For the Chinese speakers, an ORC preference was evident from the following three results: (1) the gaze duration for head nouns in ORCs was shorter than that in SRCs, (2) the gaze duration for head nouns in long-distance ORCs was shorter than that in long-distance SRCs, and (3) the regression-path duration for head nouns in ORCs was shorter than for those in SRCs. The two indicators—gaze duration and regression-path duration—can reflect the initial and later stages of sentence processing, respectively. On the other hand, although the gaze duration for embedded verbs in ORCs was longer than for those in SRCs, we noticed that the Chinese speakers skipped around 63.3% of the embedded verbs in SRCs. It seems that the components in the sentence-initial position tend to be skipped. The finding that the gaze duration for embedded verbs in ORCs was longer than for those in SRCs may be due to the skipping rate being higher for the sentence-initial embedded verbs in SRCs. Hence, the overall results of the eye-movement data from Chinese speakers indicate that ORCs were easier to process than SRCs.

As for the Japanese CSL learners, their processing pattern also demonstrated an ORC preference, which can be observed from the following five findings: (1) the gaze duration for head nouns in long-distance ORCs was shorter than that in long-distance SRCs, (2) the regression-path duration for head nouns in ORCs was shorter than for those in SRCs, (3) the regression-path duration for head nouns in long-distance ORCs was shorter than for those in long-distance SRCs, (4) the gaze duration for embedded verbs in ORCs was shorter than for those in SRCs, and (5) the regression rate for embedded verbs in ORCs was lower than for those in SRCs. These results showed that Japanese CSL learners spent a shorter time on head nouns in ORCs in the initial reading (as reflected by the shorter gaze duration) and regression process (as reflected by the shorter regression-path duration), and they spent a shorter time on embedded verbs in ORCs in the initial reading process (as reflected by the shorter gaze duration) as well as in the regression process (as reflected by the lower regression rate). Thus, these results suggest an ORC preference for the Japanese CSL learners.

In summary, the overall results from the two language groups suggest that the two groups exhibited a similar pattern in terms of processing asymmetry; that is, a tendency toward ORC preference. The finding of an ORC preference was inconsistent with the prediction of the NPAH, which proposes that it is easier to relativize the subject than the object across languages. Chinese RC processing seems to be language-specific. In addition, consider the two hypotheses (LDH and SDH), which focus on gap–filler dependencies in RCs. Specifically, the LDH proposes that the distance between a filler and its gap is determined by the linear/temporal distance, while the SDH emphasizes the role of the hierarchical phrase-structural distance between the filler and the gap. The predictions of the two hypotheses diverge in the processing asymmetry of Chinese RCs since the LDH and SDH use different methods to calculate the gap–filler distance. In other words, in the case of Chinese RC processing, the LDH would predict an ORC reference while the SDH would predict an SRC preference. Thus, our results provide solid evidence in support of the LDH.

### L1 and L2 processing of chinese RCs

In an attempt to further understand the relationship between L1 and L2 processing of Chinese RCs, we compared the processing patterns of Chinese speakers and Japanese CSL learners. In terms of processing asymmetry, both the Chinese and Japanese groups generally exhibited an ORC preference. This ORC preference in both the L1 and L2 groups supports the LDH, which indicates that the syntactic structure of Chinese plays a crucial role. On the one hand, the Chinese group exhibited an ORC preference, which can probably be attributed to the syntactic structure of RCs in Chinese, which is a head-initial language with a head-final RC construction. The tendency toward ORCs being easier to process implies that the processing difficulty can be reflected by the linear distance between the gap and the filler. In the structure of a Chinese RC, the linear distance between the gap and the filler is shorter in ORCs than in SRCs, and therefore the tendency toward an ORC preference suggests an apparent effect of linear distance on Chinese RC processing asymmetry. Our finding of an ORC preference for Chinese speakers concurs with those of previous studies (Hsiao and Gibson, 2003; Hsu and Chen, 2007; Lin and Garnsey, 2011; Gibson and Wu, 2013). On the other hand, our finding that the Japanese group also exhibited an ORC preference indicated that the syntactic structure of the target language (i.e., Chinese) was a determining factor in the RC processing. Recall that an SRC preference appears in the L1 processing of Japanese RC (Ueno and Garnsey, 2008), whereas an ORC preference was found in the L2 processing of Chinese RCs in the current study. If the NPAH—which proposes that the subject is easier to relativize than the object—holds in the RC processing of CSL, then Japanese learners will exhibit an SRC preference. Also, the SDH predicts that Japanese CSL learners will show an SRC preference since the structural distance between the gap and the filler in SRCs is shorter than in ORCs. However, the current results showed that Japanese CSL learners demonstrated an ORC preference in the processing of Chinese RCs, as did the Chinese speakers. The processing asymmetry exhibited by Japanese CSL learners was similar to that of Chinese speakers. Thus, we argue that it is the syntactic structure of the target language (i.e., Chinese) that influences how RCs are processed by Japanese CSL learners.

### The effect of modifiers

It is worth mentioning the interesting finding that both the L1 and L2 groups spent less time on the head nouns in the long-distance ORCs than on those in the short-distance ORCs. For the L1 group, the regression-path duration for the head nouns in long-distance ORCs was shorter than for those in short-distance ORCs. Also, for the L2 group, the gaze duration for the head nouns in long-distance ORCs were shorter than for those in short-distance ORCs. Together these results indicate that it was easier to process the head nouns in long-distance ORCs than those in short-distance ORCs. This finding contrasts with our expectation that the head noun in a long sentence would take a longer time to process than that in a short sentence, because in the long-distance sentence there are more antecedent elements before the head noun, which is assumed to consume more cognitive resources. Counterintuitively, it was found that the head noun in the long-distance ORCs had a shorter processing time, and we speculate that this is because the modifiers for the head nouns in long-distance ORCs can provide information to help readers predict the upcoming head nouns. The modifiers of head nouns in long-distance ORCs seem to better facilitate the processing of head nouns. This finding therefore, suggests how modifiers influence sentence processing.

## Concluding remarks

Most of the previous studies on Chinese RCs have focused on processing by L1 speakers, and few have considered the syntactic comprehension of L2 speakers. The current study employed an eye-movement technique to investigate the RC processing by Japanese CSL learners. The eye-movement data—reflecting both gaze duration and regression patterns—revealed that Japanese CSL learners and Chinese speakers have a tendency toward ORC preference. From a theoretical perspective, this ORC preference in processing Chinese RCs supports the prediction of the LDH that the key determinant of RC difficulty is the length of the gap from the head noun with which it is associated; that is, the linear distance between the gap and the filler. From an empirical perspective, these findings indicate that L1 and L2 speakers both demonstrate a tendency toward ORC preference, which suggests that the syntactic structure of RCs in Chinese is a dominating factor. The processing of Chinese RCs is language-specific in that it has a mixed pattern of a head-initial language with a head-final RC structure, constructing a shorter filler-gap distance in ORCs than that in SRCs, which perhaps makes ORCs easier to process than SRCs. In conclusion, this research expands our understanding of RC processing from L1 speakers to L2 learners of Chinese, and should provide a useful basis for further studies on the L2 processing of Chinese RCs with evidence from typologically different languages.

### Conflict of interest statement

The authors declare that the research was conducted in the absence of any commercial or financial relationships that could be construed as a potential conflict of interest.

## Footnotes

^1^Some studies on the processing of Basque RCs also showed an ORC preference (e.g., Carreiras et al., 2010), against the universal SRC preference.

^2^In (2), S, VP, CP, IP, C, and V are the subject, verb phrase, complementizer phrase, inflection phrase, complementizer, and verb, respectively.
